# Rapid discrimination of multiple myeloma patients by artificial neural networks coupled with mass spectrometry of peripheral blood plasma

**DOI:** 10.1038/s41598-019-44215-1

**Published:** 2019-05-28

**Authors:** Meritxell Deulofeu, Lenka Kolářová, Victoria Salvadó, Eladia María Peña-Méndez, Martina Almáši, Martin Štork, Luděk Pour, Pere Boadas-Vaello, Sabina Ševčíková, Josef Havel, Petr Vaňhara

**Affiliations:** 1 Department of Histology and Embryology, Faculty of Medicine, Masaryk University, Brno, Czech Republic; 2 Research Group of Clinical Anatomy, Embryology and Neuroscience (NEOMA), Department of Medical Sciences, University of Girona, Girona, Spain; 3 Experimental Neurophysiology and Clinical Anatomy (NE&AC; 2017 SGR 01279), Department of Medical Sciences, University of Girona, Girona, Spain; 4 Department of Chemistry, Faculty of Science, Masaryk University, Brno, Czech Republic; 5 Department of Chemistry, Faculty of Science, University of Girona, Girona, Spain; 6 Department of Chemistry, Analytical Chemistry Division, Faculty of Science, University of La Laguna, La Laguna, Spain; 7 Department of Clinical Hematology, University Hospital Brno, Brno, Czech Republic; 8 Department of Internal Medicine, Hematology and Oncology, University Hospital Brno, Brno, Czech Republic; 9 Babak Myeloma Group, Department of Pathological Physiology, Faculty of Medicine, Masaryk University, Brno, Czech Republic; 10 International Clinical Research Center, St. Anne’s University Hospital, Brno, Czech Republic

**Keywords:** Myeloma, Mass spectrometry, Statistics

## Abstract

Multiple myeloma (MM) is a highly heterogeneous disease of malignant plasma cells. Diagnosis and monitoring of MM patients is based on bone marrow biopsies and detection of abnormal immunoglobulin in serum and/or urine. However, biopsies have a single-site bias; thus, new diagnostic tests and early detection strategies are needed. Matrix-Assisted Laser Desorption/Ionization Time-of Flight Mass Spectrometry (MALDI-TOF MS) is a powerful method that found its applications in clinical diagnostics. Artificial intelligence approaches, such as Artificial Neural Networks (ANNs), can handle non-linear data and provide prediction and classification of variables in multidimensional datasets. In this study, we used MALDI-TOF MS to acquire low mass profiles of peripheral blood plasma obtained from MM patients and healthy donors. Informative patterns in mass spectra served as inputs for ANN that specifically predicted MM samples with high sensitivity (100%), specificity (95%) and accuracy (98%). Thus, mass spectrometry coupled with ANN can provide a minimally invasive approach for MM diagnostics.

## Introduction

Monoclonal gammopathies are a group of diseases characterized by increased amounts of abnormal immunoglobulin produced by a clone of plasma or lymphoid cells. This abnormal immunoglobulin is present in serum and/or urine in the form of entire molecule or its fragments. Monoclonal gammopathies range from benign (MGUS – monoclonal gammopathy of undetermined significance) to malignant (multiple myeloma, plasma cell leukemia, etc)^1^.

Multiple myeloma (MM) is a highly heterogeneous disease of malignant plasma cells (PC) that abrogate physiological hematopoiesis in the bone marrow; these cells are heavily dependent on the bone marrow microenvironment for survival^2^. Diagnosis of MM is currently based on: presence of monoclonal PC in the bone marrow (>10%), presence of one or more CRAB symptoms (hyperCalcemia, Renal failure, Anemia, Bone lesions) or presence of at least one biomarker of malignancy (more than 60% of clonal PC in the bone marrow, more than one bone lesion on MRI, ratio of involved/uninvolved serum light chains >100)^3^. MM comprises about 13% of hematological malignancies and around 1% of all cancers. Its incidence in Europe is 6/100 000 per year^4^. In the Czech Republic, the incidence is slightly lower, reported at 4.8/100 000 per year^5^. Median age at diagnosis is 65^6^.

Unlike other hematological malignancies that oftentimes contain a single genetic aberration that may be targeted for diagnosis, follow-up or treatment, MM is characterized by as many as seven aberrations at the time of diagnosis; this number usually increases in time. Moreover, the nature of MM makes treatment even more difficult as there are focal lesions of PC clones in the bone marrow. These clones compete for access to the bone marrow niche and evolve into a complex spatiotemporal ecosystem. At times, they may be the reason for treatment failure. Moreover, other clones may be found in various parts of the body directly influencing survival of patients^7^. In recent years, the so-called liquid biopsies, analyzing circulating cells and molecules outside of the bone marrow, are gaining in importance. In MM, bone marrow biopsy is still the golden standard for diagnosis. However, this single-site analysis may not give comprehensive information about the disease, as the clones may be located in other parts of the body^8^. Therefore, novel diagnostic approaches for detection of MM are needed.

Techniques adopted from analytical chemistry, such as mass spectrometry (MS), may provide robust and precise tools for revealing potential disease biomarkers. In cancer biology, matrix-assisted laser desorption/ionization time-of-flight (MALDI-TOF) MS is a powerful and highly sensitive tool for the detection of large numbers of peptides and proteins in serum. MS-based profiling has been applied to develop new potential biomarkers in many types of cancer, such as renal^9^, lung^10^, liver^11^, breast^12,13^, colon^14^, prostate^15^ and ovarian cancer^16^. Particularly in MM, only few studies have been conducted to find not only potential diagnostic^17–20^ and classification markers^21,22^, but also markers associated with treatment response^18^. All of them, however, require complex sample handling which increases the cost and the time to diagnosis. Thus, a different, easier and faster method is necessary.

Discrimination of disease-specific molecular patterns in peripheral blood of MM patients can provide a promising approach for early diagnostics and follow-up. Despite recent discoveries of potential low molecular mass biomarkers associated with MM, the direct applicability of metabolomics markers is still limited.

Artificial Neural Networks (ANNs) represent a mathematical tool capable of handling non-linear data and providing generalization and prediction of complex variables in multidimensional datasets. Therefore, ANNs are highly suitable for pattern recognition, classification and modelling^23^. ANNs have been widely applied in different fields of clinical diagnostics (for review see ref.^24^).

We have demonstrated previously, that ANN can recognize informative patterns in mass spectra acquired from diseased tissues^25^ or stem cells cultures^26^. In this study, we were curious whether information hidden in complex spectral patterns can provide a suitable input for ANNs to classify low mass spectral profiles of MM patients and age/sex-matched healthy donors. Such approach may represent a promising tool for diagnostics or follow up of MM patients, and also for revealing non-standard patient’s cases and/or the heterogeneity of the disease. Here we have for the first time demonstrated that MALDI-TOF MS coupled with ANNs specifically recognizes healthy and diseased samples and correctly classifies MM patients.

## Results and Discussion

**Figure 1:**
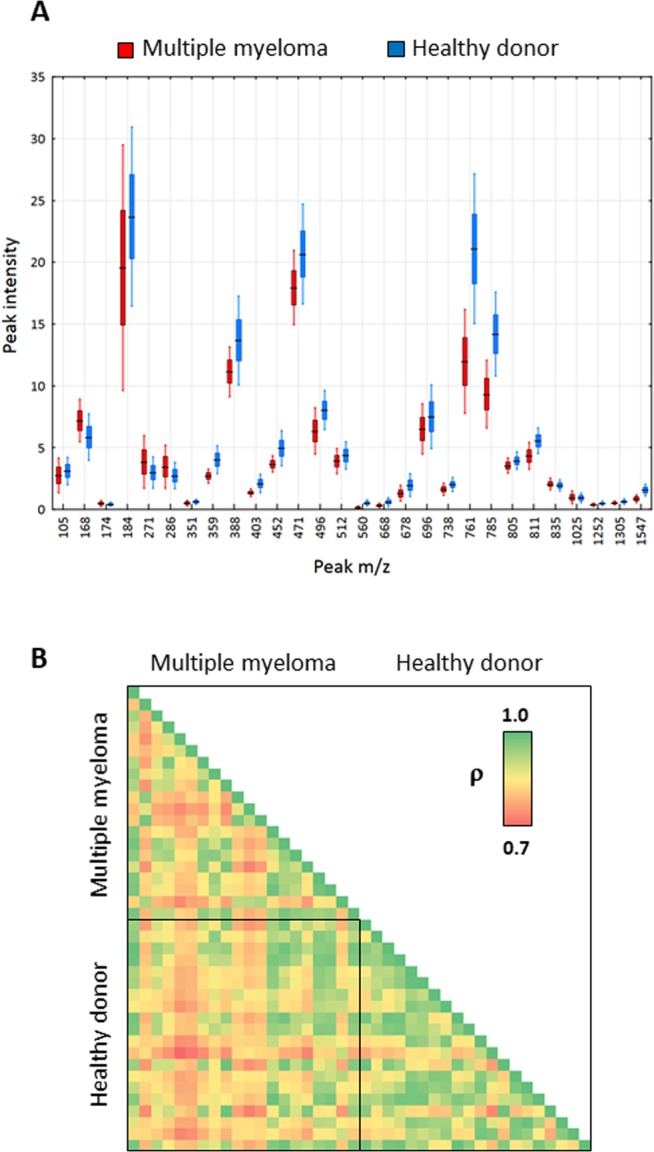
(**A**) Box and whisker plot demonstrating varying intensity of 28 peaks of distinct *m/z* in the training dataset (line: mean; box: 95% confidence intervals; whiskers: standard deviations). (**B**) Heat map of Pearson’s correlations based on spectral fingerprints in the training dataset.

**Figure 2:**
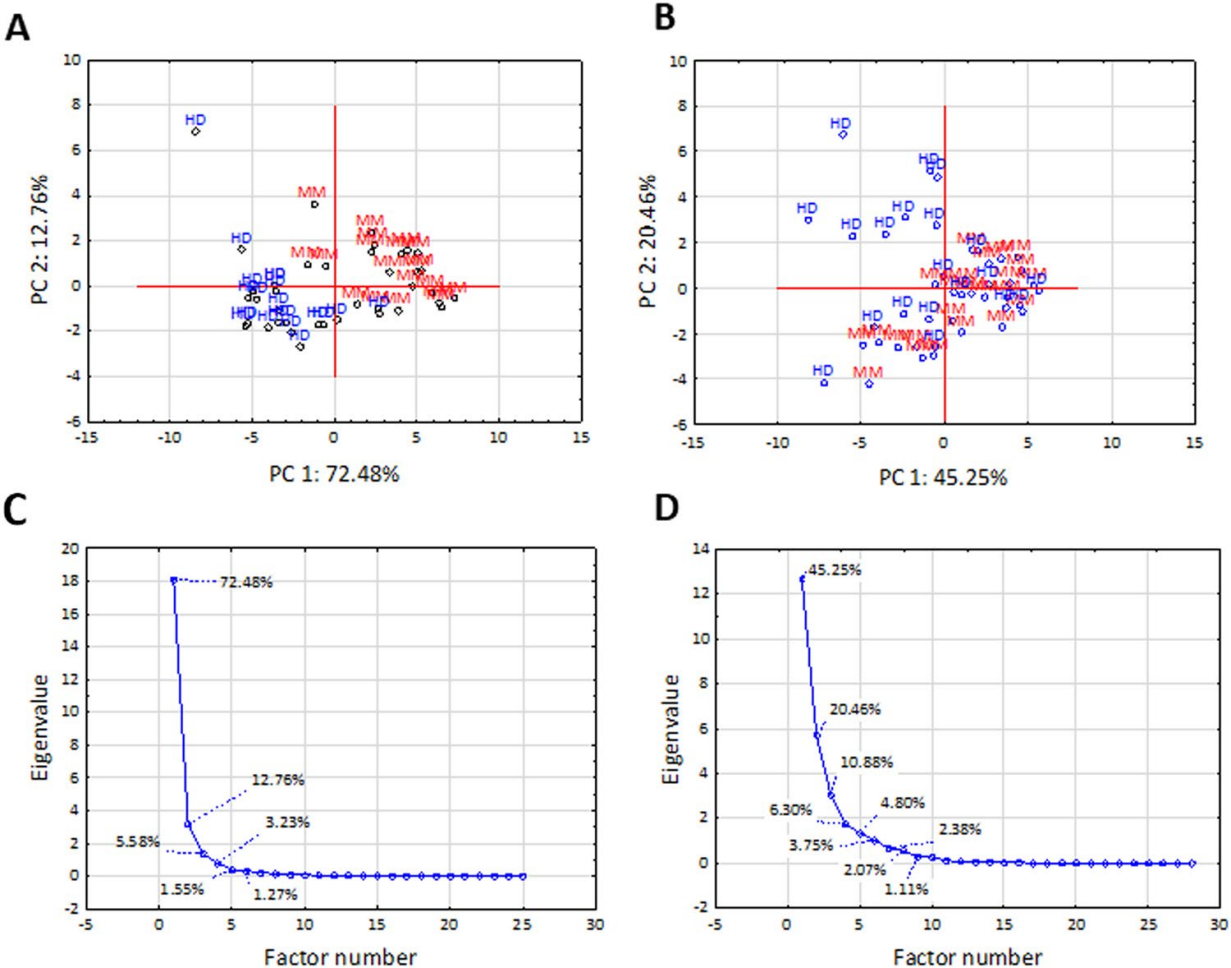
Principal component analysis of the mass spectra of the training (**A**) and validation (**B**) datasets. Each point in the PCA plot represents a unique MM patient (red) or a healthy donor (blue). Scree plot documenting the contribution of individual factors to the overall variability within the training (**C**) and validation (**D**) datasets.

We recorded mass spectra of 20 MM and 20 healthy donor (HD) samples and constructed a dataset assigned as “training”. The mass spectra were highly similar, without any apparent, constantly reproducible single peak(s) correlating to class identifiers. Rather, variable regions containing multiple peaks with lower intensity were identified. Previously, we showed that while a single-peak is either non-informative or suffers from non-linear relationships in quantitative assessments^27^, combination of several peaks may represent a more suitable input. In total, 28 peaks with varying intensities were used for calculations (Fig. 1A). First, we estimated the level of similarity in mass spectra constituting the training dataset using a heat map of Pearson’s correlations. Interestingly, the intrinsic variability of the MM cohort was higher than in HD cohort, suggesting a wider range of metabolic divergences in MM samples. Importantly, the heterogeneity between MM and HD samples was higher than within individual MM and HD datasets (Fig. 1B). This prompted us to perform principal component analysis (PCA) to discriminate the training and validation datasets (Fig. 2A,B) using the full spectral matrix as input. Eigenvalue analysis showed that most of the overall variability within the system can be explained by presence of only two factors, corresponding to MM and HD cohorts (Fig. 2C,D). Thus, we documented that spectral profiles contain sufficient information for discrimination of healthy and MM samples in a single coherent dataset.

**Figure 3:**
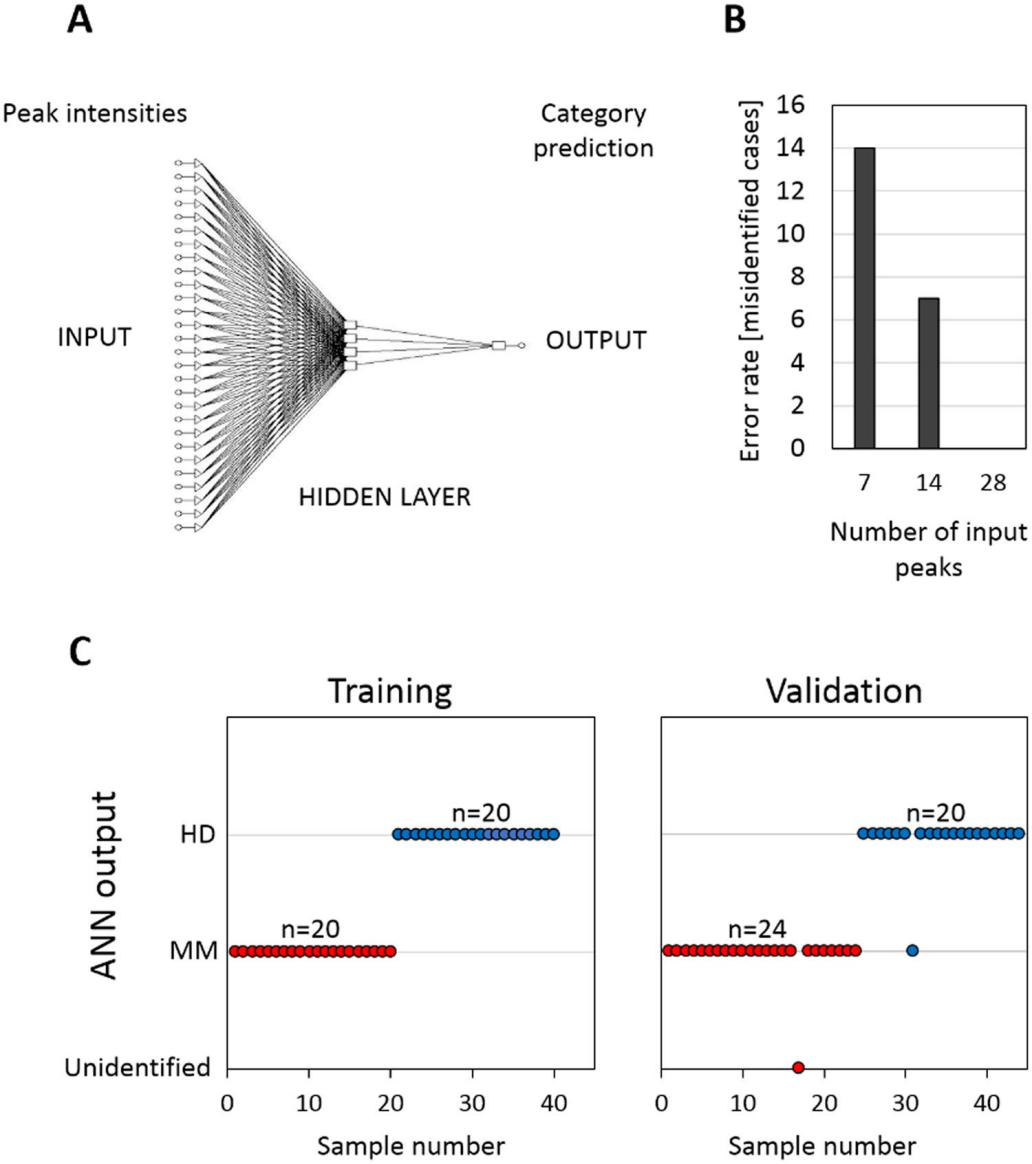
(**A**) Architecture of the artificial neural network used for the prediction of sample class (MM, HD). (**B**) Plot documenting prediction capability of the ANN based on 7, 14 or 28 input peaks. (**C**) Plots documenting ANN classification outputs in the training and validation dataset.

To independently validate the observations, we constructed a back-propagation multilayer perceptron ANN (Fig. 3A). The optimal network architecture was validated by plotting the Root Mean Square Error (RMS) against the number of nodes (data not shown). The robustness was demonstrated by performing >50,000 training cycles (epochs), without significant overfitting the ANN model. First, the leave-one-out cross-validation method was used to test the neural network to predict single cases within the training dataset with 100% of correct predictions. However, when we randomly decreased number of peaks in the spectral matrix entering the analysis, the error rate was significantly increased (Fig. 3B). This suggests that the dataset lacks a dominant “marker peak“ associated with sample class, and the discriminating capacity of mass spectra is dependent on the cumulative contributions of alterations in more molecular species. Thus, the non-reduced mass spectrum can provide a global marker for prediction of MM.

Then, we established the validation dataset consisting of additional 24 MM and 20 HD samples measured independently on the same instrument. While the PCA correctly clustered the MM and HD cohorts within the validation dataset, it failed in case of merged training and validation datasets. Interestingly, the inter-experimental variability introduced a significant bias to the analysis, as the PCA clustered the merged dataset according to the experiment (event of measurement) suffering from the sensitivity to systematic errors (data not shown). When the trained ANN was used to predict classification for the independent validation dataset, 42 cases out of 44 were correctly classified as HD or MM (Fig. 3C, Table 1).

**Table 1:** Results of ANN classification.

Dataset	Training	Verification
Cases	40 (20 MM, 20 HD)	44 (24 MM, 20 HD)
True positive (MM)	20 (100%)	23 (95.83%)
True negative (HD)	20 (100%)	19 (95.00%)
False positive	0	1 (5.00%)
False negative	0	0
Unidentified	0	1 (4.17%)*
Sensitivity [%]	100%	95.83%
Specificity [%]	100%	95.00%
Accuracy [%]	100%	95.45%

*Unidentified case (MM) arbitrarily classified as false negative.

In concordance to the training set, the validation ANN analysis was dependent on the number of input signals, confirming the initial observations (data not shown). Similar results were obtained when we recapitulated the study on a different MALDI-TOF MS instrument (AXIMA Resonance). In summary, we demonstrated for the first time that peripheral blood plasma of MM patients contains specific spectral fingerprints corresponding to alterations of low mass molecular species, which might be used for disease prediction or revealing druggable metabolic pathways.

Several studies suggested that metabolomics is a new and powerful tool usable for identification of early biomarkers related with inborn disorders of metabolism, cardiovascular diseases and cancer. This approach allows for analysis of small molecules that characterize metabolic pathways. Generally, two approaches have been used so far: nuclear magnetic resonance spectroscopy (H-NMR) and mass spectrometry (MS). Using H-NMR, Puchades Carrasco *et al*.^18^ showed differences in metabolomics profiles of MM patients (at diagnosis and CR) versus healthy donors. This study showed that while MM patients at diagnosis had a different metabolomics profile in comparison to healthy donors, once they reached CR, they had closer metabolic profiles to healthy donors. A newer study by Medriano *et al*.^28^ used liquid chromatography coupled to mass spectrometry to assess metabolomics in MM and non-Hodgkin lymphoma patients in comparison to healthy donors. They found a clear metabolic difference in samples of cancer patients in comparison to healthy donors. The observed affected pathways, such as oxidative phosphorylation and choline metabolism, were those linked to tumor growth and progression. Another study using compared metabolomics profiles of samples of MGUS, MM patients (both at diagnosis and at relapse) and found that all three groups of patients had significantly different profiles from healthy donors. Pathway analyses revealed a distinct metabolic profile with significant alterations in amino acid, lipid, and energy metabolism in healthy donors compared to MGUS/MM patients^29^.

MALDI-TOF MS–driven approach in MM was first suggested by Wang *et al*. in 2008. In their work, the authors identified 56 unique *m/z* signals ranging from *m/z* 2015 to 24070, with varying intensities between MM and healthy donors and demonstrated that three of them are of a potential prognostic significance^19^. In our work, we revealed potentially informative spectral regions in lower *m/z* values (<1000 Da) of peripheral blood plasma and documented that they can serve as suitable inputs for reliable ANN-based diagnostics. In conclusion, we envisage that disease related spectral fingerprints coupled with artificial intelligence can provide a complementary, minimally-invasive tool for diagnostics and follow-up of MM patients.

## Material and Methods

### Chemicals

Sinapinic acid, trifluoroacetic acid, and Micro-90^®^ concentrated cleaning solution were purchased from Sigma-Aldrich (Prague, Czech Republic). Acetonitrile (purity for isotachophoresis) was purchased from Merck (Darmstadt, Germany). Red phosphorus was purchased from Riedel de Haën (Hannover, Germany). Water was double distilled using a quartz apparatus from Heraeus Quarzschmelze (Hanau, Germany). All other reagents were of analytical grade purity. IVD bacterial test standard (BTS) was purchased from Bruker Daltonik GmbH (Bremen, Germany).

### Sample collection and preparation

In total, 84 samples of plasma of peripheral blood acquired from 44 MM patients and 40 age- and sex-matched HD, were included in the analysis (Table 2). The total cohort was randomly divided into the training and validation dataset consisting of 20 MM and 20 HD samples, and 24 MM and 20 HD samples, respectively. The training and validation sets were processed and measured independently. All MM patients were diagnosed at the University Hospital Brno, Czech Republic and included into the study only after they signed the informed consent form approved by the Ethics committee of the University Hospital Brno, and Ethics committee of the Faculty of Medicine, Masaryk University, in accordance with the current version of the Helsinki Declaration. All patients’ samples were collected at the time of diagnosis prior to treatment. Peripheral blood was stabilized in EDTA, centrifuged at 2000 g for 15 min at 20 °C. Aliquoted plasma samples were stored at −80 °C and thawed only once prior to analysis.

**Table 2:** Experimental cohorts entering the analysis.

	Group	*n*	Gender	Age median (min-max) [years]
**Training cohort**	Healthy donor	20	M = 10 F = 10	67 (51–66)
	Multiple myeloma	20	M = 10 F = 10	67 (57–83)
**Validation cohort**	Healthy donor	20	M = 10 F = 10	56 (54–64)
	Multiple myeloma	24	M = 12 F = 12	71 (47–95)

### Sample preparation for MS

Samples of plasma were thawed and processed on ice. All samples were diluted ten times with double distilled water and mixed in 1:1 ratio with solution of sinapinic acid (SA) containing 20 mg/ml SA in 50% acetonitrile supplemented with 0.3% trifluoroacetic acid. 1 μl of the mixture was spotted on purified stainless steel target plate in three replicates. To avoid carry-over contamination, the target plate was regularly cleaned with specific cleaning procedure in an ultrasonic bath with ultrapure solvents sequentially in this order: methanol, acetone, 5% Micro-90, water, methanol, water and then dried before spotting the samples.

### Acquisition of mass spectra

Mass spectra were acquired using Axima CFR with Time-Of-Flight (TOF) analyzer and validated using AXIMA Resonance TOF mass spectrometer from Kratos Analytical Ltd. (Manchester, UK). Both devices are equipped with a nitrogen laser (337 nm), delayed extraction, and microchannel plate detector. The laser energy was expressed in arbitrary units (scale 0 to 180 a.u.). The laser power and fluence at the maximum laser energy were 6 mW and ≈10 mJ/mm^2^/pulse, respectively. The accelerating voltage was set to 20 kV and laser repetition to 5 Hz. All measurements were carried out at 10^−4^ Pa pressure in the TOF tube. Mass spectra obtained in ranges *m/z* 0–10 000 from 2500 laser shots using regular raster are displayed as the ion signal to the *m/z* value. The spectra were normalized meaning that the intensity of the maximum peak was equal to 100%. Calibration was performed using phosphorus clusters as described elsewhere^30^ or a bacterial test standard with an accuracy of 0.01 Da. The Launchpad software (Kompact version 2.3.4, 2003) from Kratos Analytical Ltd. was used to evaluate and export the mass spectra to ASCII. The smoothing of mass spectra by Savitzky-Golay method, baseline subtraction by LOESS method, and spectral alignment, were performed in the R-program^31^. Only reproducible peaks with intensities over the signal:noise >3 were included to the analysis. In total, 28 informative peaks with minimal inter-experimental variability, and sufficient variability between cohorts, were selected from all technically available peaks.

### Statistical analysis

Pearson’s correlations, principal component analyses (PCA) and determination of eigenvalues were done in Statistica 6.0 (StatSoft, Inc., Tulsa, USA). Artificial neural networks were constructed and run in TRAJAN Neural Network Simulator, Release 3 (Trajan Software Ltd., Durham, UK)^32^. The ANN contained 28 input nodes corresponding to the number of peak intensities, four nodes in the hidden layer and the output layer. The network was trained using conjugate gradient descent training algorithm, with maximum number of iterations 50,000. Classification confidence level was set to 0.05. Values of continuous variables over 0.95 or below 0.05 were considered as the decision outputs to a discrete class. Values between 0.05 and 0.95 were classified as unknown. Cases that were not identified by ANN (decision output “unknown”) were arbitrarily classified as wrong predictions in the respective cohort.

### Ethics statement

All experiments were performed in accordance with relevant guidelines and regulations. Informed consent for study participation was obtained for each patient included. The study was approved by the Ethics committee of the University Hospital Brno (approval date February 2, 2017), and by the Ethics committee of the Faculty of Medicine, Masaryk University (approval date June 22, 2016, no.: 21/2016).

## Data Availability

The datasets generated during and/or analyzed during the current study are available from the corresponding author upon request.
